# Can e-commerce mitigate the negative impact of COVID-19 on international trade?

**DOI:** 10.1007/s42973-021-00099-3

**Published:** 2021-09-29

**Authors:** Kazunobu Hayakawa, Hiroshi Mukunoki, Shujiro Urata

**Affiliations:** 1 Bangkok Research Center, Institute of Developing Economies, 127 Gaysorn Tower, Ratchadamri Road, Lumphini, Pathumwan, Bangkok, 10330 Thailand; 2 Faculty of Economics, Gakushuin University, Mejiro 1-5-1, Toshima-ku, Tokyo 171-8588 Japan; 3 Development Studies Center, Institute of Developing Economies, 3-2-2 Wakaba, Mihama-ku, Chiba-shi, Chiba 261-8545 Japan

**Keywords:** COVID-19, E-commerce, Trade, F15, F53

## Abstract

This study aims to empirically investigate the role of E-commerce (EC) on the trade impacts of COVID-19. To this end, we estimate gravity equations for bilateral trade among 34 reporting countries and their 145 partner countries during January–August in 2019 and 2020. Our major findings can be summarized as follows. A larger number of confirmed cases or deaths in both importing and exporting countries significantly decrease international trade. However, we found that EC development in importing countries contributes to mitigating this negative effect of COVID-19 on trade while that in exporting countries does not. These results are robust for our use of multiple measures of EC development.

## Introduction

E-commerce (EC) is getting increasingly popular since the outbreak of the coronavirus disease 2019 (hereafter, COVID-19) pandemic. Due to the lockdown policy, also known as “stay-at-home orders,” people have not been permitted to leave the house with the exception of getting daily exercise, going grocery shopping, and making “essential” trips. Thus, to do their shopping while avoiding possible COVID-19 infection, consumers started to purchase items online. As a result, the EC market has been growing dramatically all over the world. For example, in Japan in 2020, sales in the three largest EC markets (Rakuten, Amazon, and Yahoo) rose by 7% in January, 13% in February, and 14% in March from the same month the preceding year, according to a report by Nint, Inc. Specifically, EC sales of medical products (e.g., masks) have exploded. This explosive growth of EC markets can be observed globally. According to *eMarketer*, while worldwide retail sales are expected to decrease by 3% in 2020, retail EC sales are predicted to increase by 28%.

EC business is expected to mitigate the negative effects of COVID-19 on international trade. Online shopping is not limited to domestic transactions. It is also possible in international transactions, known as cross-border EC. While staying at home, consumers can order various products including imported items. Therefore, if the EC market develops in a country, the negative impact of COVID-19 on import demand will decrease in that country. In addition, EC enables firms to conduct business-to-business (B2B) transactions online and search for new suppliers and customers despite supply chain disruption. Thus, without traveling abroad or attending international business exhibitions, firms may discover new business partners online. This B2B EC scheme is expected to mitigate the negative impact of COVID-19 on export supply. In short, EC business will play a key role in international trade during this pandemic period.

This study aims to empirically investigate the role of EC on the trade impacts of COVID-19. Due to a lack of reliable data on international transactions via EC, we examine how the harmful effects of COVID-19 on trade differ depending on the availability of EC. To this end, we estimate gravity equations for bilateral trade among 34 reporting countries and their 145 partner countries during the period of January–August in 2019 and 2020. We introduce two kinds of variables in our estimation. One captures the extent of COVID-19’s damage (e.g., the number of deaths) in exporting and importing countries. The estimated coefficient for this variable indicates the average effect of COVID-19’s damage on international trade. The other is an interaction term of the COVID-19 variable with the variable indicating the development of EC business in the respective country. For the latter variable, we use the *readiness* of EC business, which indicates how prepared an economy is to support online shopping. The coefficient for this interaction term shows how such EC business readiness in each country changes the effect of COVID-19’s damage on trade.

Our major findings can be summarized as follows. A higher number of confirmed COVID-19 cases or deaths in both importing and exporting countries significantly decrease international trade. However, we found that EC development mitigates this negative effect of COVID-19 on trade in importing countries while it does not do so in exporting countries. These results are robust for our use of multiple measures of EC development. The insignificant contribution of exporters’ EC may indicate that exporters have not adopted EC business and that it may take some time before they can use EC for international transactions. This contrast between exporting and importing countries may indicate that cross-border business-to-consumer (B2C) EC business is more developed and widely used than cross-border B2B EC business.

The number of studies on EC is growing in not only the management science literature but also in the field of economics. Anson et al. (2019) investigated exchange rate pass-through in online shopping. The influence of distance or virtual proximity on cross-border EC has been examined by Hortaçsu et al. (2009), Gomez-Herrera et al. (2014), Hellmanzik et al. (2015), and Lendle et al. (2016).^1^ These studies consistently found that geographical distance mattered not only in offline transactions but also in online transactions. Chang and Meyerhoefer (2021) examined the effect of COVID-19 on online food shopping in Taiwan. They discovered that an additional confirmed case of COVID-19 increased online sales by 5.7% and the number of online customers by 4.9%. This finding indirectly supports our expectation that online shopping mitigates the negative effect of COVID-19 on international trade.

Our study is also related to the literature on the international trade–COVID-19 nexus. Fuchs et al. (2020) empirically investigated whether previous economic linkages established through trade and investment as well as political relations were associated with China’s export pattern of critical medical goods. Hayakawa and Mukunoki (2021a, 2021b, 2021c) investigated the effects of COVID-19 on worldwide trade. Like Chang and Meyerhoefer (2021), Hayakawa and Mukunoki (2021a, 2021c) examined how the number of confirmed COVID-19 cases in addition to that of COVID-19 deaths affected international trade or global value chains. Hayakawa and Mukunoki (2021b) also investigated if the existence of lockdown policies, such as stay-at-home and workplace-closing orders, reduced international trade by employing monthly trade data. These studies established the negative effects of COVID-19, particularly in exporting countries. Against this backdrop, we empirically investigate how EC businesses change such negative effects of COVID-19.

The remainder of this study is organized as follows. Section 2 summarizes our conceptual framework regarding the relationship between COVID-19’s effects on trade and EC. After explaining the empirical framework used to examine this relationship in Sect. 3, we report our estimation results in Sect. 4. Section 5 concludes.

## Conceptual framework

This section provides a conceptual framework about how COVID-19 affects trade between countries and how EC mitigates the possible negative impact of COVID-19. The spread of infectious diseases and lockdown policies influence both the demand and supply sides of the economy. On the demand side, COVID-19 damage such as disease and death decreases aggregate demand by reducing people’s earnings. Lockdown policies and social distancing lower people’s mobility in retail, and they shrink the markets for non-essential products that are durable and “postpone-able” (Baldwin and Tomiura, 2020). Decreased demand for non-essential products reduces imports of those products, although the spread of COVID-19 is likely to increase imports of essential products such as food and medical supplies.

On the supply side, the disease and death of people due to COVID-19 decrease the workforce. Lockdown measures and social distancing decrease people’s mobility in workplaces. These shocks reduce the supply of products and exports. Moreover, the suspension of production activities, delivery delays of input materials, and a decline in maritime transportation have caused supply chain disruptions and decreased supply. Although the introduction of a telecommuting system would underpin firms’ activities, the lack of physical interaction might decrease productivity, reducing supply further. A negative shock on productivity discourages firms from entering export markets, as indicated by Melitz (2003). Furthermore, the ban on overseas business trips makes it challenging to obtain information about foreign customers, increasing market penetration costs and decreasing exports, as suggested by Arkolakis (2010).

The development of EC business will help mitigate the negative impact on both the demand side as well as the supply side. On the demand side, B2C EC business gives consumers options for purchasing products online without visiting brick-and-mortar retail stores. Watanabe and Omori (2020) found that online consumption of goods and services has been increasing while offline consumption has been decreasing during the COVID-19 pandemic. The opportunity to sell products online and deliver them directly to consumers prevents producers’ and retailers’ earnings from decreasing. Thus, the availability of EC in importing countries is expected to mitigate the negative effect of COVID-19 on import demand. We call this effect *the demand-preserving effect* of EC business.

On the supply side, the development of B2B EC (e.g., online exhibitions or online catalogs) will play a key role in mitigating supply disruptions. Garicano and Kaplan (2001) have suggested that B2B EC reduces coordination costs between buyers and sellers because searching for products, sellers, and prices will be less costly if conducted online. Thus, under supply disruptions, B2B EC will help producers match new intermediates and service suppliers. The role of B2B EC in finding new partners is pivotal in improving firm performance. Lee et al. (2003) classified B2B EC into “basic B2B EC” and “collaborative B2B EC.” Basic B2B EC represents the B2B networks with the primary role of receiving and sending orders online. Collaborative B2B EC represents the B2B networks that help establish new opportunities for collaboration with network partners. Based on survey data, Lee et al. (2003) showed that collaborative B2B EC improves firm performance while basic B2B EC has a limited role. Therefore, B2B EC that establishes new links between buyers and sellers will mitigate supply shocks and weaken the negative effect of COVID-19 on export supply. We call this effect *the supply-preserving effect* of EC business.

## Empirical framework

Based on the conceptual framework explained in the previous section, this section presents the empirical framework that we use to investigate the impact of COVID-19 on trade. The basic model is specified as follows.

1 $${Trade}_{ijym}=\mathrm{exp}\left\{{\alpha }_{1}{COVID}_{iym}+{\beta }_{1}{COVID}_{jym}+{\delta }_{ijy}+{\delta }_{ijm}+{\delta }_{ym}\right\}\cdot {\epsilon }_{ijym}$$

${Trade}_{ijym}$ is the export value from country *i* to *j* in month *m* of year *y*. As explained in more detail later, ${COVID}_{iym}$ and ${COVID}_{jym}$ represent the extent of COVID-19 damage in exporting and importing countries, respectively. We control for three kinds of fixed effects (${\delta }_{ijy}$, ${\delta }_{ijm}$, and ${\delta }_{ym}$). ${\epsilon }_{ijt}$ is a disturbance term. Country–pair year fixed effects (${\delta }_{ijy}$) control for standard gravity variables such as geographical distance in addition to the effects of trade agreements, importer’s demand sizes, and exporter’s factor prices (e.g., wages). Country–pair month fixed effects (${\delta }_{ijm}$) control for the seasonality of trade between the two countries. ${\delta }_{ym}$ denotes year-month fixed effects, which control for global shocks.

In Eq. (1), we capture the average effect of COVID-19 on trade using the coefficients for the *COVID* variables. We extend this model to examine the role of EC business. Specifically, our estimation equation is modified as follows.

2 $${Trade}_{ijym}=\mathrm{exp}\left\{{\alpha }_{1}{COVID}_{iym}+{\alpha }_{2}{COVID}_{iym}\times {EC}_{i}+{\beta }_{1}{COVID}_{jym}+{\beta }_{2}{COVID}_{jym}\times {EC}_{j}+{\delta }_{ijy}+{\delta }_{ijm}+{\delta }_{ym}\right\}\cdot {\epsilon }_{ijym}$$

In this extended model, we add the interaction terms of the *COVID* variables with the development of EC business (*EC*). The coefficient for the interaction term indicates how the impact of COVID-19 on trade differs according to the level of EC development. While the non-interacted terms of the *COVID* variables are expected to have negative coefficients, the demand-preserving and supply-preserving effects of EC business discussed in Sect. 2 will produce positive coefficients for the interacted terms. We estimate this equation using the Poisson pseudo maximum likelihood (PPML) method.

Our data sources are as follows. The study covers trade from January to August in 2019 and 2020. We obtain monthly data on both exports and imports in the reporting countries for these periods from the *Global Trade Atlas* maintained by the IHS Markit.^2^ The export values are included in the dataset after multiplying them by 1.05 to roughly adjust for freight and insurance charges. Our dataset consists of trade values among 34 reporting countries and their 145 partner countries, which are listed in Appendix A.^3^ As a measure of COVID-19 damage, we use the number of COVID-19 cases and deaths, data on which are obtained from the *European Centre for Disease Prevention and Control*.^4^ Specifically, we use the sum of the number of new cases and deaths in each month for 2020. The numbers are set to zero for 2019.^5^ While these numbers indicate the extent of physical damage, a high number of cases or deaths discourages even uninfected persons from working or going outside. Thus, we expect these numbers to be quite related to the economic conditions caused by COVID-19.

The more challenging issue lies in the variable for EC business because of data limitations. One of the measures is the *readiness* of EC businesses. For example, the *UNCTAD B2C E-commerce Index 2019* (hereafter, the EC index) is available from the United Nations Conference on Trade and Development (UNCTAD) website.^6^ According to its explanation, a higher EC index value indicates that an economy is more prepared to support online shopping. The index consists of four indicators in 2018 that are highly related to online shopping.^7^ The UNCTAD also shows that the extent to which people shop online in a country is correlated with the value of the index, with an adjusted R-squared value of 0.8. In the baseline analysis, we use this index. In the later analysis, we also use the share of EC retail sales out of total retail sales, which indicates the *outcome* or *magnitude* of EC business.

**Fig. 1:**
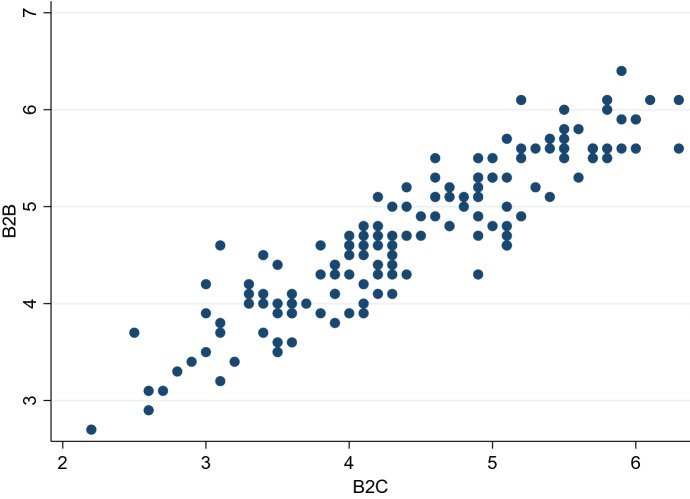
Correlation between B2B Internet Use and B2C Internet Use. *Source*: The Global Information Technology Report, 2015. *Note*: The correlation coefficient is 0.91

The EC index indicates to what extent people can use online services to shop. Thus, the interaction term of this index with COVID-19 in an importing country is related to the role of the readiness or availability of the B2C market in the trade impact of COVID-19. Namely, its coefficient shows the demand-preserving effect. Although the EC index basically covers B2C EC, we assume that it is positively correlated to B2B EC. This assumption seems reasonable because countries with a large B2C EC market tend to have a large B2B EC market. Indeed, as shown in Fig. 1, according to the Global Information Technology Report 2015 by the World Economic Forum and *Institut Européen d ‘Administration des Affaires* (INSEAD), B2B Internet use is positively and highly correlated with B2C internet use among 143 countries.^8^ Thus, with the EC index, we expect the coefficient for the interaction term with COVID-19 in exporting countries to show the supply-preserving effect. In short, both interaction terms are expected to have positive coefficients.

**Fig. 2:**
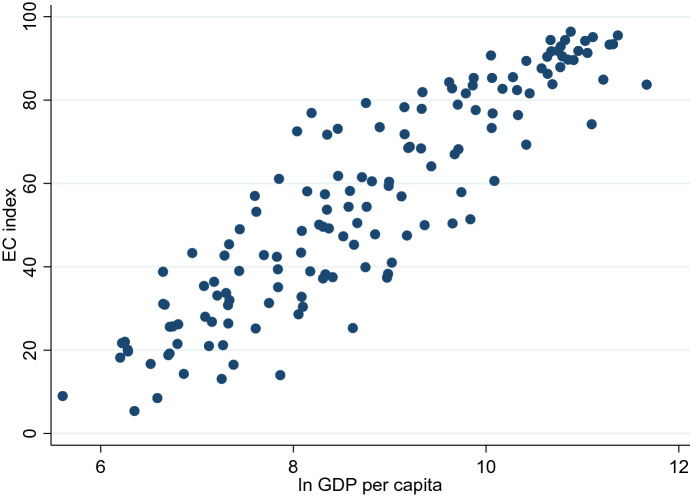
Correlation between EC Index and Logged GDP per capita. *Sources*: UNCTAD B2C E-commerce Index, 2019; World Development Indicators. *Notes*: We use GDP per capita in 2018. The correlation coefficient is 0.90

Table 1 shows the top 10 and bottom 10 countries in the EC index. Those data are available for 152 countries. Most of the top 10 countries are European countries. The Netherlands is the top-ranking country, followed by Switzerland. The United States, China, and Japan, which have big EC markets, are not listed here. Thus, we should keep in mind that this index reflects the readiness of EC businesses rather than their size. In contrast, Afghanistan is the worst country in terms of the readiness of EC businesses, followed by Liberia. Overall, the EC index seems to take a higher value for countries with a higher income. Indeed, as shown in Fig. 2, the EC index is positively correlated with logged GDP per capita in 2018.

**Table 1:** The EC index

Best 10 countries		Worst 10 countries	
	EC index		EC index
Netherlands	96.4	Niger	5.4
Switzerland	95.5	Chad	8.5
Singapore	95.1	Burundi	9.0
Finland	94.4	Comoros	13.1
United Kingdom	94.4	Dem. Rep. of the Congo	13.8
Denmark	94.2	Congo	14.0
Norway	93.4	Guinea	14.3
Ireland	93.3	Mauritania	16.5
Germany	92.9	Liberia	16.7
Australia	91.8	Afghanistan	18.2

*Source*: UNCTAD B2C E-commerce Index, 2019

## Empirical results

This section reports our estimation results for Eq. (2). The basic statistics for our variables are listed in Table 2. In this table, we also report the statistics for non-interacted EC variables. The sample mean, minimum, and maximum are, respectively, around 0.67, 0.05, and 0.96. Table 3 reports the estimation results. We cluster standard errors by country–pair. Columns (I) and (II) use the number of cases and deaths for the *COVID* variables, respectively. The coefficients for both importer’s and exporter’s *COVID* variables are significantly negative in terms of both the number of cases and deaths. These results indicate that the damage caused by COVID-19 in both exporting and importing countries has a negative effect on trade, as found by Hayakawa and Mukunoki (2021a). The coefficients for the interaction terms with the EC variable for importers are estimated to be significantly positive in terms of both the number of cases and deaths. On the export side, on the other hand, only the interaction term with the number of confirmed cases has a significantly positive coefficient.

**Table 2:** Basic statistics

	Obs	Mean	Std. Dev	Min	Max
Trade	128,628	1.6E+08	19.31E+08	0	4.2E+10
ln (1 + Imp cases)	128,628	3.097	4.173	0	14.500
ln (1 + Imp cases) * Imp EC	128,628	2.147	3.186	0	13.202
Imp EC	128,628	0.663	0.251	0.054	0.964
Imp Stay	128,628	0.271	0.424	0	1
Imp Stay * Imp EC	128,628	0.178	0.303	0	0.964
ln (1 + Exp cases)	128,628	3.103	4.177	0	14.500
ln (1 + Exp cases) * Exp EC	128,628	2.166	3.195	0	13.202
Exp EC	128,628	0.670	0.246	0.054	0.964
ln (1 + Imp deaths)	128,599	1.612	2.674	0	10.965
ln (1 + Imp deaths) * Imp EC	128,599	1.137	2.047	0	10.011
ln (1 + Exp deaths)	128,601	1.617	2.674	0	10.965
ln (1 + Exp deaths) * Exp EC	128,601	1.146	2.051	0	10.011

*Sources*: Authors’ computation

*Note*: “Trade” is measured in US dollar

**Table 3:** Estimation results of Eq. (2)

	(I)	(II)	(III)	(IV)	(V)	(VI)
ln (1 + Imp COVID)	− 0.041*** [0.004]	− 0.054*** [0.006]	− 0.016*** [0.002]	− 0.014*** [0.003]	− 0.039*** [0.008]	− 0.063*** [0.011]
ln (1 + Imp COVID) * 1 for Imp L					0.013 [0.015]	0.023 [0.022]
ln (1 + Imp COVID) * 1 for Imp LM					− 0.001 [0.005]	0.000 [0.007]
ln (1 + Imp COVID) * 1 for Imp UM					− 0.001 [0.003]	0.005 [0.005]
ln (1 + Imp COVID) * Imp EC	0.037*** [0.006]	0.055*** [0.009]	0.034** [0.017]	0.025 [0.016]	0.036*** [0.009]	0.065*** [0.012]
ln (1 + Exp COVID)	− 0.033*** [0.005]	− 0.033*** [0.006]	− 0.017*** [0.003]	− 0.022*** [0.004]	− 0.030*** [0.011]	− 0.034** [0.015]
ln (1 + Exp COVID) * 1 for Exp L					0.022 [0.014]	0.025 [0.023]
ln (1 + Exp COVID) * 1 for Exp LM					− 0.003 [0.005]	− 0.004 [0.007]
ln (1 + Exp COVID) * 1 for Exp UM					− 0.001 [0.004]	0.001 [0.006]
ln (1 + Exp COVID) * Exp EC	0.012** [0.006]	0.012 [0.007]	− 0.054** [0.023]	− 0.009 [0.017]	0.009 [0.012]	0.012 [0.016]

Ecom measure	Index	Index	Share	Share	Index	Index
COVID measure	Cases	Deaths	Cases	Deaths	Cases	Deaths
Log pseudolikelihood	− 1E+11	− 1E+11	− 1E+11	− 1E+11	− 1E+11	− 1E+11
Pseudo *R*^2^	0.9972	0.9972	0.9971	0.9971	0.9972	0.9972
No. of obs.	128,628	128,516	128,628	128,516	127,548	127,436

This table reports the estimation results obtained using the PPML method. ***, **, and * indicate, respectively, the 1%, 5%, and 10% levels of statistical significance. The standard errors reported in parentheses are those clustered by country pair. In all specifications, we control for country pair–year fixed effects, country pair–month fixed effects, and year-month fixed effects. “COVID measure” indicates the measure of the COVID-19 variables. “Cases” and “Deaths” represent the number of confirmed cases and deaths, respectively. “Ecom measure” indicates the variable on the EC. For “Index,” we use the UNCTAD B2C E-commerce Index 2019, while we employ the share of retail EC sales out of total retail sales in 2019 for “Share.” For income class, “L,” “LM,” and “UM” indicate low income, lower-middle income, and upper-middle income, respectively

As shown in Table 1, higher-income countries seem to have a higher EC index value. Therefore, it might be interesting to compute the average coefficient for the COVID-19 variable according to income level. To do that, we use the income classification adopted by the World Bank. Each country is categorized as either high-income, upper-middle-income, lower-middle-income, or low-income. Specifically, we computed the simple average of EC values of the countries recorded as importing (exporting) countries, and the resultant average EC index values are as follows, 0.870 (0.869) for high-income countries, 0.598 (0.603) for upper-middle-income countries, 0.401 (0.407) for lower-middle-income countries, and 0.209 (0.212) for low-income countries. Combining these average values with the estimates in column (I), the average coefficient for importer’s COVID-19 cases becomes − 0.009 for high-income countries, − 0.019 for upper-middle-income countries, − 0.026 for lower-middle-income countries, and − 0.033 for low-income countries. The corresponding coefficients for exporter’s COVID-19 cases are − 0.023, − 0.026, − 0.028, and − 0.030. Thus, due to the development of EC environment, the higher-income countries have the smaller negative effect of COVID-19.

We conduct three kinds of robustness checks on the results above. First, although we prefer the readiness measure of EC, we also try to use the market size of EC. Specifically, we use the share of retail EC sales out of total retail sales in 2019, data for which are obtained from *eMarketer*.^9^ This variable is not a direct measure of EC size because it is normalized by total retail sales and is thus related to the readiness of EC transactions, to some extent. Nevertheless, this measure is based on the actual (i.e., not the potential) size of EC transactions in the pre-pandemic period. Therefore, we examine this variable for a robustness check by interacting it with the *COVID* variables. The results are presented in Columns (III) and (IV). Non-interacted terms of the *COVID* variables again have significantly negative coefficients. While the coefficients for the interaction term for importers are significantly positive, the interaction term for exporters has negative coefficients for both the number of cases and deaths.^10^

Second, we further control for the heterogeneous effects of COVID-19 according to the level of economic development of each country. As shown in Table 1, higher-income countries seem to have a higher EC index value. There are various reasons why the negative effects of COVID-19 are lower in high-income countries. For example, the hygienic environment is better in high-income countries than in low-income countries. We introduce the interaction terms of the *COVID* variables with the dummy variables on the income groups to which the countries belong. The income groups are again based on the classification by the World Bank, including high-income, upper-middle-income, lower-middle-income, and low-income groups. In the estimation, the base category is set to high-income countries.

Columns (V) and (VI) report the results. The significantly negative coefficients for the (non-interacted) *COVID* variables imply negative effects for high-income exporters and importers. All interaction terms with dummy variables on income groups have insignificant coefficients. Thus, there are no significant differences in the trade effects of COVID-19 according to a country’s income class. Non-high-income countries also suffer from a similar magnitude of negative effects as those observed in high-income countries. As is consistent with the results in the previous columns, the interaction terms with the EC index again have significantly positive coefficients for importers but not for exporters.

These results indicate that EC development in importing countries contributes to mitigating the negative effect of COVID-19 on trade while that in exporting countries does not. Namely, we discovered a demand-preserving effect of EC business but not a supply-preserving effect. B2C EC business plays a key role in the result for importing countries. Although the cross-border B2C EC market has grown since the pre-pandemic period, more consumers seem to have switched their shopping patterns from in-person to online during the pandemic period. In contrast, the insignificant result in exporting countries may indicate that cross-border B2B business is still in a transitional period. Online business discussion or matching is becoming popular because business travel is restricted in most countries. It will take more time for the transactions in the form of cross-border B2B business to grow in international trade.

Another reason for the insignificant result in exporting countries might be attributable to the reduction of domestic production. The spread of infectious diseases and lockdown measures cause labor shortages, decrease productivity, and thereby decrease domestic production.^11^ The decreased production reduces exports, and this reduction cannot be mitigated by cross-border B2B business. New health regulations, movement controls, and the reduced demands for air flights reduce cross-border transport and logistics services, and the disruptions in international transportation affect the exports of all goods that require physical delivery, including those purchased through B2B EC.^12^

Furthermore, our use of the B2C EC index for B2B EC might be a reason for the insignificant result in exporting countries. Because of this possible problem, we drop the exporter-side trade effects and focus on the importer side and further examine the robustness of the results for importing countries. Specifically, we estimate the following equation.

3 $${Trade}_{ijym}=\mathrm{exp}\left\{{\beta }_{1}{COVID}_{jym}+{\beta }_{2}{COVID}_{jym}\times {EC}_{j}+{\delta }_{ijy}+{\delta }_{ijm}+{\delta }_{iym}\right\}\bullet {\epsilon }_{ijym}$$

All exporter-specific effects, including the effects of COVID-19, are absorbed by exporter–year-month fixed effects (${\delta }_{iym}$). The estimation results are shown in Table 4. We use two types of EC variables and control for the heterogeneous effects of COVID-19 according to the economic development level of the importing country. Similar to the results in Table 3, the coefficients for the importer’s COVID-19 variable are significantly negative, while its interaction terms with the EC index have significantly positive coefficients (except for Column (IV)).^13^^,^^14^

**Table 4:** Estimation results for importers: Eq. (3)

	(I)	(II)	(III)	(IV)	(V)	(VI)
ln (1 + Imp COVID)	− 0.044*** [0.004]	− 0.062*** [0.006]	− 0.018*** [0.002]	− 0.013*** [0.003]	− 0.041*** [0.007]	− 0.059*** [0.011]
ln (1 + Imp COVID) * 1 for Imp L					0.011 [0.014]	0.019 [0.021]
ln (1 + Imp COVID) * 1 for Imp LM					− 0.001 [0.005]	− 0.003 [0.006]
ln (1 + Imp COVID) * 1 for Imp UM					− 0.002 [0.003]	− 0.001 [0.004]
ln (1 + Imp COVID) * Imp EC	0.041*** [0.006]	0.064*** [0.008]	0.039** [0.016]	0.013 [0.017]	0.038*** [0.008]	0.062*** [0.012]

Ecom measure	Index	Index	Share	Share	Index	Index
COVID measure	Cases	Deaths	Cases	Deaths	Cases	Deaths
Log pseudolikelihood	− 1E+11	− 1E+11	− 1E+11	− 1E+11	− 1E+11	− 1E+11
Pseudo R2	0.9976	0.9976	0.9976	0.9976	0.9976	0.9976
No. of obs.	128,628	128,570	128,628	128,570	128,088	128,030

This table reports the estimation results obtained using the PPML method. ***, **, and * indicate, respectively, the 1%, 5%, and 10% levels of statistical significance. The standard errors reported in parentheses are those clustered by country pair. In all specifications, we control for country pair–year fixed effects, country pair–month fixed effects, and exporter–year-month fixed effects. “Covid measure” indicates the measure of the COVID-19 variables. “Cases” and “Deaths” represent the numbers of confirmed cases and deaths, respectively. “Ecom measure” indicates the variable on the EC. For “Index,” we use the UNCTAD B2C E-commerce Index 2019, while we employ the share of retail EC sales out of total retail sales in 2019 for “Share.” For income class, “L,” “LM,” and “UM” indicate low income, lower-middle income, and upper-middle income, respectively

Last, as another robustness check on the results for importing countries, we use an alternative measure of COVID-19. Specifically, we use the share of days in each month when stay-at-home orders were effective in the importing countries. We obtained the necessary information from the Oxford COVID-19 Government Response Tracker (OxCGRT) (Hale et al. 2020). The OxCGRT systematically collects information on several different common policy responses that the governments of more than 160 countries have taken to respond to the pandemic using 17 indicators. The measure for importing countries was constructed using “C6 Stay-at-home requirements,” which includes “1—recommends not leaving home,” “2—requires not leaving home, with exceptions for daily exercise, grocery shopping, and ‘essential’ trips,” and “3—requires not leaving home with minimal exceptions (e.g., allowed to leave once a week, only one person can leave at a time, etc.).” Regardless of the degree of the severity of the orders, we counted the number of days when a score of at least one was registered and used its share out of the total number of days in each month. We label this measure “*Imp Stay*.”

The results are shown in Table 5. As in the previous tables, we use the two kinds of EC variables and control for the heterogeneous effects of COVID-19 according to the level of economic development of the importing countries. We discovered that the coefficients for *Imp Stay* are significantly negative, which indicates that the longer the period of stay-at-home orders in importing countries is, the smaller the volume of international trade becomes. Its interaction term with the EC variables has significantly positive coefficients, as is consistent with the results presented in the previous tables. In sum, we conclude that EC development in the importing countries contributes to mitigating the negative effects of COVID-19 on trade.

**Table 5:** An alternative COVID measure: Eq. (3)

	(I)	(II)	(III)
Imp Stay	− 0.363*** [0.038]	− 0.091*** [0.016]	− 0.435*** [0.074]
Imp Stay * 1 for Imp L			0.176 [0.107]
Imp stay * 1 for Imp LM			0.051 [0.041]
Imp Stay * 1 for Imp UM			0.014 [0.030]
Imp Stay * Imp EC	0.380*** [0.048]	0.521*** [0.111]	0.459*** [0.081]

Ecom measure	Index	Share	Index
Log pseudo likelihood	− 1.2E+11	− 1.2E+11	− 1.2E+11
Pseudo *R*^2^	0.9976	0.9975	0.9976
No. of obs.	128,628	128,628	128,088

This table reports the estimation results obtained using the PPML method. ***, **, and * indicate, respectively, the 1%, 5%, and 10% levels of statistical significance. The standard errors reported in parentheses are those clustered by country pair. In all specifications, we control for country pair–year fixed effects, country pair–month fixed effects, and exporter–year-month fixed effects. “Ecom measure” indicates the variable on the EC. For “Index,” we use the UNCTAD B2C E-commerce Index 2019, while we employ the share of retail EC sales out of total retail sales in 2019 for “Share.” For income class, “L,” “LM,” and “UM” indicate low income, lower-middle income, and upper-middle income, respectively. “Imp stay” indicates the share of days when stay-at-home orders were effective in importing countries

## Concluding remarks

In this paper, we showed that EC in importing countries mitigates the negative impact of COVID-19 on international trade. With the prospect of a prolonged COVID-19 pandemic, the possible future emergence of new infectious diseases, and the advancement of digital transformation, EC is likely to increase in importance in domestic and international transactions. Indeed, EC can play a significant role in contributing to the growth of the global economy by promoting globalization via expanded trade. To achieve healthy growth in EC, the establishment of a rules-based, free, open, stable, and transparent environment for conducting EC business is important. While the rules on EC have been constructed as a part of many free trade agreements, such as the Comprehensive and Progressive Trans-Pacific Partnership agreement and the EU–Japan Economic Partnership Agreement, an international rule or agreement has not yet been established. In December 2017, 71 WTO members declared that they would initiate exploratory work toward future WTO negotiations on digital trade. Since then, several meetings have been held with additional members, but negotiations have not yet begun. A number of challenges must be handled before negotiations can even start. It is hoped that negotiations begin and are concluded soon so that international EC business will grow rapidly, contributing to overall economic growth.

## Appendix A. Study countries

34 Reporting Countries (ISO 3-letter codes):

ARG, AUS, AUT, BEL, BRA, CAN, CHE, CHN, CIV, DEU, DNK, ESP, FRA, GBR, GRC, HKG, IDN, IRL, ISR, JPN, KEN, KOR, LUX, MEX, MYS, NLD, PHL, PRT, RUS, SGP, SWE, THA, USA, ZAF.

145 Partner Countries (ISO 3-letter codes):

AFG, AGO, ALB, ARE, ARG, AUS, AUT, AZE, BDI, BEL, BEN, BFA, BGD, BGR, BHR, BIH, BLR, BLZ, BOL, BRA, BTN, BWA, CAN, CHE, CHL, CHN, CIV, CMR, COG, COL, COM, CRI, CYP, CZE, DEU, DJI, DNK, DOM, DZA, ECU, EGY, ESP, EST, ETH, FIN, FRA, GAB, GBR, GEO, GHA, GIN, GRC, GTM, HKG, HND, HRV, HTI, HUN, IDN, IND, IRL, IRN, IRQ, ISL, ISR, ITA, JAM, JOR, JPN, KAZ, KEN, KGZ, KHM, KOR, KWT, LAO, LBN, LBR, LBY, LKA, LSO, LTU, LUX, LVA, MAR, MDA, MDG, MEX, MLI, MMR, MNG, MOZ, MRT, MUS, MWI, MYS, NAM, NER, NGA, NIC, NLD, NOR, NPL, NZL, OMN, PAK, PAN, PER, PHL, POL, PRT, PRY, QAT, ROM, RUS, RWA, SAU, SDN, SEN, SGP, SLE, SLV, SVK, SVN, SWE, SYR, TCD, TGO, THA, TJK, TTO, TUN, TUR, TZA, UGA, UKR, URY, USA, UZB, VEN, VNM, YEM, ZAF, ZMB, ZWE.

## Appendix B. Other tables

**Table B1:** Controlling for GDP per capita: Eq. (3)

	(I)	(II)
ln (1 + Imp COVID)	− 0.058*** [0.012]	− 0.091*** [0.016]
ln (1 + Imp COVID) * Imp EC	0.027*** [0.010]	0.034*** [0.011]
ln (1 + Imp COVID) * ln Imp GDP per capita	0.002 [0.002]	0.005** [0.002]

COVID measure	Cases	Deaths
Log pseudolikelihood	− 1E+11	− 1E+11
Pseudo *R*^2^	0.9976	0.9976
No. of obs	127,120	127,062

This table reports the estimation results obtained using the PPML method. ***, **, and * indicate, respectively, the 1%, 5%, and 10% levels of statistical significance. The standard errors reported in parentheses are those clustered by country pair. In all specifications, we control for country pair–year fixed effects, country pair–month fixed effects, and exporter–year–month fixed effects. “Covid measure” indicates the measure of the COVID-19 variables. “Cases” and “Deaths” represent the numbers of confirmed cases and deaths, respectively. As an EC measure, we use the UNCTAD B2C E-commerce Index 2019

**Table B2:** Estimation Results of Eq. (3) by Industry

	Cases		Deaths	
		* Imp EC		* Imp EC
Live animals	− 0.013	0.010	− 0.014	0.014
Vegetable products	0.017	− 0.022	0.02	− 0.028
Animal/vegetable fats and oils	0.008	− 0.014	− 0.002	0.005
Food products	− 0.036***	0.029***	− 0.051***	0.050***
Mineral products	− 0.025**	0.009	− 0.040**	0.030
Chemical products	− 0.011**	0.003	− 0.020***	0.016
Plastics and rubber	− 0.053***	0.047***	− 0.073***	0.073***
Leather products	− 0.094***	0.061***	− 0.115***	0.096***
Wood products	− 0.032***	0.024**	− 0.047***	0.048***
Paper products	− 0.044***	0.041***	− 0.052***	0.056***
Textiles and apparels	− 0.089***	0.104***	− 0.110***	0.151***
Footwear	− 0.061***	0.027***	− 0.082***	0.062***
Plastic or glass products	− 0.028***	0.003	− 0.043***	0.026**
Precious metals	− 0.072**	0.109**	− 0.153***	0.223***
Base Metal	− 0.045***	0.025***	− 0.068***	0.055***
Machinery	− 0.044***	0.040***	− 0.051***	0.052***
Transport equipment	− 0.081***	0.066***	− 0.111***	0.106***
Precision machinery	− 0.032***	0.020***	− 0.031***	0.023**
Miscellaneous	− 0.062***	0.041***	− 0.082***	0.069***

This table reports the estimation results obtained using the PPML method. Specifically, we estimate the models in columns (I) and (II) in Table 3 by industry (HS Section classification). ***, **, and * indicate, respectively, the 1%, 5%, and 10% levels of statistical significance, which are based on the standard errors clustered by country pair

## Footnotes

1 Examples of other studies on EC include Einav et al. (2014), Biswas and Kennedy (2016), Cavallo (2017), and Dolfen et al. (2019).

2 See https://connect.ihsmarkit.com/gta/home.

3 One issue is that trade values are not separately available between January and February in 2020 when China is a reporting country. Thus, we do not use those data in both 2019 and 2020.

4 See https://data.europa.eu/euodp/en/data/dataset/covid-19-coronavirus-data.

5 The use of their ratio to total population did not change our results by much because we controlled for country–pair fixed effects.

6 See https://unctad.org/en/PublicationsLibrary/tn_unctad_ict4d14_en.pdf.

7 The index is calculated as the average of four indicators (i.e., each indicator carries the same weight) using data for 2018 or the latest available. Those include account ownership at a financial institution or with a mobile money service provider, individuals using the Internet postal reliability index (drawn from the Universal Postal Union), and secure Internet servers.

8 B2B Internet use is based on the question, “In your country, to what extent do businesses use ICTs for transactions with other businesses?” Similarly, for B2C Internet use, the question is, “In your country, to what extent do businesses use the Internet for selling their goods and services to consumers?” A score is assigned to each question ranging from zero (not at all) to seven (to a great extent). The correlation coefficient between these two scores is 0.91.

9 The top 10 countries include China, the United Kingdom (UK), South Korea, Denmark, Norway, the US, Finland, Sweden, Japan, and France.

10 The negative and significant coefficient of the interaction term for exporters in Column (III) might be because the development of EC facilitated the domestic supply of scarce goods amid supply disruptions, reducing the export supply of the goods. Besides that, the share of EC transactions might reflect the basic B2B EC rather than the collaborative B2B EC. As is discussed in Sect. 2, the basic B2B EC has a limited role in mitigating the supply shock. Furthermore, our use of retail EC sales can be a reason for the unexpected result because it might not correspond to the size of B2B transactions.

11 According to the interview surveys conducted by JETRO in Singapore, Thailand, Malaysia, Indonesia, and the Philippines, many Japanese manufacturing firms experienced production reductions, a decline in productivity, and faced difficulties in exporting due to disruptions in international transportations. See www.jetro.go.jp/biz/areareports/special/2020/0901/d4fb00237115fc57.html (written in Japanese).

12 See the WTO report (https://www.wto.org/english/tratop_e/covid19_e/ecommerce_report_e.pdf) and Part 2 of Chapter 2 in UNCTAD (2020) for details.

13 Notice that some results show the larger absolute magnitude in the interacted variable than in the non-interacted variable. However, this result does not mean the positive effect of COVID-19 damages. For example, in column (II), the sample average of EC index is 0.663. Thus, in a country with the average index, the resulting coefficient for the number of COVID-19 deaths becomes − 0.020 (= − 0.062 + 0.064 * 0.663). Similarly, in column (III), the resulting coefficient in countries with the average EC share becomes − 0.016 (= − 0.018 + 0.039 * 0.054).

14 The results are unchanged when introducing the interaction term of the COVID variable with logged GDP per capita rather than the interaction term with dummy variables on income groups. The results are shown in Table B1 in Appendix B. In addition, we estimate the models in columns (I) and (II) in Table 4 by industry (19 industries in total), and the results are shown in Table B2 in Appendix B. The interaction term with the number of cases has a significantly positive coefficient in 13 industries. In particular, the absolute magnitude is relatively large in the textiles and apparels industry and the precious metals industry. On the other hand, the interaction term is insignificant in agricultural goods.

## References

[CR1] Anson J, Boffa M, Helble M (2019). Consumer arbitrage in cross-border e-commerce. Review of International Economics.

[CR2] Arkolakis C (2010). Market penetration costs and the new consumers margin in international trade. Journal of Political Economy.

[CR3] Baldwin, R., & Tomiura, E. (2020). Thinking ahead about the trade impact of COVID-19. In R. Baldwin & B.W. di Mauro (Eds.), *Economics in the Time of COVID-19* (pp. 59–71). Centre for Economic Policy Research.

[CR5] Biswas, T., & Kennedy, P. L. (2016). The effect of the internet on bilateral trade. 2016 Annual Meeting, February 6–9, 2016, San Antonio, Texas 229994, Southern Agricultural Economics Association.

[CR6] Cavallo A (2017). Are online and offline prices similar? Evidence from large multi-channel retailers. American Economic Review.

[CR7] Chang H-H, Meyerhoefer CD (2021). COVID-19 and the demand for online food shopping services: Empirical evidence from Taiwan. American Journal of Agricultural Economics.

[CR8] Dolfen, P., Einav, L., Klenow, P. J., Klopack, B., Levin, J. D, Levin, L., & Best, W. (2019). Assessing the gains from e-commerce. NBER Working Paper No. 25610. National Bureau of Economic Research.

[CR9] Einav L, Levin J, Popov I, Sundaresan N (2014). Growth, adoption, and use of mobile e-commerce. American Economic Review.

[CR10] Fuchs A, Kaplan L, Kis-Katos K, Schmidt SS, Turbanisch F, Wang F (2020). Mask wars: China’s exports of medical goods in times of COVID-19. Covid Economics.

[CR11] Garicano L, Kaplan SN (2001). The effects of business-to-business e-commerce on transaction costs. Journal of Industrial Economics.

[CR12] Gomez-Herrera E, Martens B, Turlea G (2014). The drivers and impediments for cross-border e-commerce in the EU. Information Economics and Policy.

[CR13] Hale T, Webster S, Petherick A, Phillips T, Kira B (2020). Oxford COVID-19 Government Response Tracker.

[CR14] Hayakawa K, Mukunoki H (2021). Impacts of COVID-19 on international trade: Evidence from the first shock. Journal of the Japanese and International Economies.

[CR15] Hayakawa K, Mukunoki H (2021). Impacts of lockdown policy on international trade. Asian Economic Papers.

[CR16] Hayakawa K, Mukunoki H (2021). Impacts of Covid-19 on global value chains. The Developing Economies.

[CR17] Hellmanzik C, Schmitz M (2015). Virtual proximity and audiovisual services trade. European Economic Review.

[CR18] Hortaçsu A, Martínez-Jerez F A, Douglas J (2009). The geography of trade in online transactions: Evidence from eBay and MercadoLibre. American Economic Journal: Microeconomics.

[CR19] Lee SC, Pak BY, Lee HG (2003). Business value of B2B electronic commerce: the critical role of inter-firm collaboration. Electronic Commerce Research and Applications.

[CR20] Lendle A, Olarreaga M, Schropp S, Vézina P-L (2016). There goes gravity: eBay and the death of distance. Economic Journal.

[CR23] Melitz MJ (2003). The impact of trade on intra-industry reallocations and aggregate industry productivity. Econometrica.

[CR21] UNCTAD, 2020, *COVID-19 and E-Commerce: A Global Review*, Geneva: The United Nation, Available at: https://unctad.org/webflyer/covid-19-and-e-commerce-global-review.

[CR22] Watanabe T, Omori Y (2020). Online consumption during the COVID-19 crisis: Evidence from Japan. Covid Economics.

